# Prospective in-patient cohort study of moves between levels of therapeutic security: the DUNDRUM-1 triage security, DUNDRUM-3 programme completion and DUNDRUM-4 recovery scales and the HCR-20

**DOI:** 10.1186/1471-244X-12-80

**Published:** 2012-07-13

**Authors:** Mary Davoren, Sarah O'Dwyer, Zareena Abidin, Leena Naughton, Olivia Gibbons, Elaine Doyle, Kim McDonnell, Stephen Monks, Harry G Kennedy

**Affiliations:** 1 National Forensic Mental Health Service, Central Mental Hospital, Dundrum, Dublin 14, Ireland; 2 Department of Psychiatry, Trinity College, Dublin, Ireland

**Keywords:** Risk, Violence, Forensic, Secure hospital, Moves, DUNDRUM, HCR-20

## Abstract

**Background:**

We examined whether new structured professional judgment instruments for assessing need for therapeutic security, treatment completion and recovery in forensic settings were related to moves from higher to lower levels of therapeutic security and added anything to assessment of risk.

**Methods:**

This was a prospective naturalistic twelve month observational study of a cohort of patients in a forensic hospital placed according to their need for therapeutic security along a pathway of moves from high to progressively less secure units in preparation for discharge. Patients were assessed using the DUNDRUM-1 triage security scale, the DUNDRUM-3 programme completion scale and the DUNDRUM-4 recovery scale and assessments of risk of violence, self harm and suicide, symptom severity and global function. Patients were subsequently observed for positive moves to less secure units and negative moves to more secure units.

**Results:**

There were 86 male patients at baseline with mean follow-up 0.9 years, 11 positive and 9 negative moves. For positive moves, logistic regression indicated that along with location at baseline, the DUNDRUM-1, HCR-20 dynamic and PANSS general symptom scores were associated with subsequent positive moves. The receiver operating characteristic was significant for the DUNDRUM-1 while ANOVA co-varying for both location at baseline and HCR-20 dynamic score was significant for DUNDRUM-1. For negative moves, logistic regression showed DUNDRUM-1 and HCR-20 dynamic scores were associated with subsequent negative moves, along with DUNDRUM-3 and PANSS negative symptoms in some models. The receiver operating characteristic was significant for the DUNDRUM-4 recovery and HCR-20 dynamic scores with DUNDRUM-1, DUNDRUM-3, PANSS general and GAF marginal. ANOVA co-varying for both location at baseline and HCR-20 dynamic scores showed only DUNDRUM-1 and PANSS negative symptoms associated with subsequent negative moves.

**Conclusions:**

Clinicians appear to decide moves based on combinations of current and imminent (dynamic) risk measured by HCR-20 dynamic score and historical seriousness of risk as measured by need for therapeutic security (DUNDRUM-1) in keeping with Scott's formulation of risk and seriousness. The DUNDRUM-3 programme completion and DUNDRUM-4 recovery scales have utility as dynamic measures that can off-set perceived 'dangerousness'.

## Background

The decision to move a forensic mental health patient from conditions of high to medium to low security is one of the most important decisions taken by forensic mental health professionals but has seldom been studied. Risk assessment has evolved into structured professional judgement instruments which guide decision makers by identifying risk factors for violence or suicide but do not claim to make 'diagnostic' ratings for risk status. The DUNDRUM toolkit is a suite of structured professional judgement instruments developed at the Central Mental Hospital Dundrum, Dublin, Ireland [1]. The toolkit is a set of instruments designed to assist clinical decision making when assessing the need for therapeutic security (DUNDRUM-1 triage security) [2] and urgency of need for therapeutic security (DUNDRUM-2 triage urgency) [3], assessing a patient's programme completion (DUNDRUM-3) and recovery (DUNDRUM-4) and therefore their ongoing need for security as well as a self rated instrument in which patients assess their own need for therapeutic security [1].

The content of these instruments is different from but complementary to risk assessment. When making decisions regarding moving a patient from high to medium and on to low levels of therapeutic security or discharging patients to the community, clinicians are likely to take more than risk assessment alone into account. Factors such as mental health, physical health, self care and activities of daily living, family and social networks, use of leave from the hospital and other such factors are all given strong consideration. These items are often included in clinician’s reports to mental health tribunals and review boards to assist these bodies in their decision making with regard to a patient’s readiness for a move to a less secure place. The items scored in the DUNDRUM-3 and DUNDRUM-4 include the above items and are based on motivation theory, cycle of change and engagement. The DUNDRUM-3 programme completion instrument consists of seven items - physical health, mental health, drugs and alcohol, problem behaviours, self care and activities of daily living, occupation, education and creativity and family and social networks. The six items of the DUNDRUM-4 recovery scale are stability, insight, rapport and working alliance, leave, HCR-20 dynamic risk items and victim sensitivity. Each item is accompanied by a series of definitions and rated from ‘0’ to ‘4’. A patient scoring mostly ‘4’s is unlikely to be ready for a move to a less secure place, a patient scoring mainly ‘3’s is likely to be ready to move from high to medium security, mainly ‘2’s is likely to be ready to move from medium security to PICU, mainly ‘1’s is ready for placement in the community and mainly ‘0’s is likely to be ready for an absolute discharge.

We have shown that these scales have excellent psychometric properties and in a naturalistic cross-sectional study of patients in a forensic hospital, these instruments distinguished between those who are or are not allowed unaccompanied leave and between those who have progressed to pre-discharge units [4].

### Rationale

These instruments are designed to enable clinicians to present assessments regarding the readiness for moves to less secure places in a way that is transparent and evidence based. The content of the instruments is derived from an iterative process [1-4]. The factors making up the items are broader than risk assessment, although there is good evidence that risk assessment is relevant to the success or failure of moves from higher to lower levels of therapeutic security [5-8]. The examination of the decision making process leading to success or failure in the placement is a therapeutic departure from the more commonly studied outcomes such as violence or criminal recidivism [8,9] and addresses long standing problems with revolving door patients and the need for reliable international benchmarks [10]. The DUNDRUM Toolkit instruments can be used in ways that are complimentary to risk assessment instruments when engaging with service users in planning their treatment and when presenting evidence at mental health review tribunals and boards concerning detention under mental health legislation, when measuring outcomes for health service commissioners and for service users.

### Objectives

In this prospective study we examined whether the DUNDRUM-1, DUNDRUM-3 and DUNDRUM-4 along with other assessment instruments could distinguish between those who subsequently moved from more secure to less secure units, and those who were moved from less secure to more secure units. Because the HCR-20 is established as a predictor of moves and recalls [5-8], we hypothesised that any other measure would have to have a significant effect even when correcting for HCR-20 dynamic risk. We further hypothesised that the risk of violence or self-harm, combined with the seriousness of the risk, would be significant determinants of the decisions to move patients, based on the original formulation of Scott concerning risk and the seriousness of the risk [11]. We hypothesised that the need for therapeutic security, as measured by the DUNDRUM-1 triage security instrument, and the DUNDRUM-3 programme completion and DUNDRUM-4 recovery instruments, which record progress, would represent Scott's concept of 'seriousness' or 'gravity'. In a subsequent paper, we will report on whether these instruments predicted those who were eventually conditionally discharged.

## Method

### Study design

This is a naturalistic twelve month prospective cohort study. Data was gathered as part of the clinical audit of service delivery. The study was approved by the research ethics, audit and effectiveness committee of the Central Mental Hospital. All participants were given a written account of the nature and purpose of the study and all participants gave their informed consent.

### Setting

The Central Mental Hospital Dundrum is the only secure forensic psychiatric hospital in the Republic of Ireland. It provides high, medium and low secure therapeutic wards on a single campus serving a population of 4.6 million. Patients are detained either under the Mental Health Act (2001) or the Criminal Law (Insanity) Act 2006 [12]. Male patients in the Central Mental Hospital are admitted to a high secure admission ward. From there they progress to a series of medium secure units and finally to low secure and pre-discharge units. Patients are moved from more secure wards to less secure wards along this recovery pathway. This system is patient centred as each patient is placed at an appropriate level of therapeutic security according to their individual need. These placements correspond to levels of risk, symptom severity and the patient’s overall level of functioning [13-15]. Location at the time of assessment (location at baseline) was ranked according to the level of therapeutic security. This is an ordinal ranking according to the staff-to-patient (S:P) ratio in whole-time equivalents for ward-based staff: the high secure intensive care unit = 10 (n = 6 patients, 19 whole time equivalent staff, staff to patient (S:P) ratio 3.2:1), the admission high secure unit = 9 (n = 12, S:P ratio 2.2:1), the first medium secure unit = 8 (n = 16, S:P ratio 1.5:1), the second medium secure unit = 7 (n = 16, S:P ratio 1.4:1), a longer term low secure unit (locked) = 6 (n = 9, S:P ratio 1.2:1), a low secure pre-discharge unit = 5 (n = 15, S:P ratio 0.9:1), a hospital hostel ward = 4 (n = 9, S:P ratio 0.8:1), the community high support residence = 3 (n = 6, S:P ratio 1.2:1), a total of 91 places with 100% bed occupancy. These rankings match the position of each unit on the recovery pathway as patients progress through the hospital from admission to discharge. The longer term low secure unit and the high secure intensive care units are optional placements according to assessed need, while the 'main' pathway through care proceeds from the high secure admission unit through the first and then second medium secure units to the pre-discharge unit then to the hospital hostel ward and on to the high support community residence. Patients may however be discharged from any point in the recovery pathway if appropriate or if they come to the end of a fixed sentence. Decisions regarding moves along the recovery pathway were made at a weekly transfers and referrals meeting. During the period of this study these decisions were made based on unstructured professional judgment.

All in-patients during the month of February 2010 were eligible. Data on subsequent moves were then gathered up to 31st March 2011. For this analysis the 8 women patients were excluded as their recovery pathway is different from the recovery pathway for the men patients.

### Variables: outcome measures

The patient movements were documented up to 31st March 2011, an observation period of 1.07 years. A 'positive' move was recorded if there was any move from a unit ranked at a higher level of security to a unit ranked less secure. A 'negative' move was recorded if there was any move from a less secure unit to a unit ranked more secure.

### Variables: measurement instruments

All those eligible were assessed by MD and SO'D using the DUNDRUM-1 triage security instrument, DUNDRUM-3 programme completion instrument and DUNDRUM-4 recovery instrument [1], in order to assess the patient's recovery and readiness for a move to less secure places. In order to test for confounding or biasing factors, all patients were also assessed for the Positive and Negative Symptom Score (PANSS) [16], to assess the severity of positive, negative and general symptoms of schizophrenia and mental illness where higher scores indicate more severe symptoms. The Global Assessment of Function (GAF) measured the patient's social, psychological and occupational functioning on a scale from 0 to 100, where 100 is perfect functioning [17] assessed by LN & OG. The HCR-20 (The historical, clinical and risk management-20) was used to rate each patient's risk of violence[18] and S-RAMM (suicide risk assessment and management manual) in order to rate risk of suicide [14] performed by the treating clinicians, collated by LN & OG. The Camberwell Assessment of Need Forensic Version (CANFOR) clinician rated unmet needs [19] is designed to identify the needs of forensic patients and this was rated by treating clinicians and collated by KMcD.

### Confounding and bias

Possible sources of confounding and bias were considered, including the need for therapeutic security at baseline (measured by the DUNDRUM-1 triage security instrument [1,2]) since this might influence clinicians to be more conservative when deciding to move a patient to a less secure unit. Risk of violence was measured by the HCR-20 [18] and risk of self harm and suicide was measured by the S-RAMM [20]. Measures of mental state (PANSS) [16,21] and global function (GAF) [17,21] and staff-rated unmet need for care (CANFOR) [19] were also assessed as possible confounders.

### Study size

In this naturalistic audit study, based on the previous year we estimated that there would be approximately 12 positive moves over the course of a year. Examining the largest cross-sectional differences between units (levels of therapeutic security) in Table 1 we calculated that this number would be sufficient to detect an effect size of 1.0 or greater for the DUNDRUM-1, DUNDRUM-3 and DUNDRUM-4.

**Table 1:** Location and baseline measures

	**SABU**	**Male admission**	**MMSU1**	**MMSU2**	**LTLSU**	**rehab**	**Hostel ward**	**24 hour Nurse Care**	**ANOVA F**	**ANOVAP value**
**n**	**6**	**10**	**14**	**14**	**10**	**15**	**11**	**6**	**Df = 7**	
DUNDRUM-1	32.7(28.4-36.9)	26.8(22.9-30.7)	31.2(28.4-34.0)	31.6(29.0-34.2)	31.0(29.0-33.0)	28.5(25.8-31.3)	27.7(24.8-30.6)	25.5(23.0-27.9)	2.9	0.009
DUNDRUM-3	23.0(18.4-27.7)	21.4(18.9-23.9)	21.0(18.5-23.5)	17.8(16.0-19.6)	18.6(14.6-22.6)	12.8(10.1-15.5)	7.7(4.1-11.4)	5.3(0.0-11.6)	17.7	0.001
DUNDRUM-4	20.3(17.9-22.8)	18.4(16.0-20.8)	20.3(18.6-21.9)	19.1(17.9-20.4)	19.7(17.7-21.7)	13.8(10.9-16.7)	7.9(5.9-9.9)	7.3(0.0-15.0)	19.6	0.001
GAF	35.3(17.8-52.9)	45.8(35.3-56.3)	44.9(37.5-52.4)	55.0(49.6-60.4)	47.8(40.3-55.3)	60.2(56.4-63.9)	69.6(62.9-76.2)	72.5(55.0-89.9)	10.0	0.001
PANSS pos	20.3(10.9-29.8)	13.6(11.1-16.1)	19.2(14.9-23.5)	13.0(9.9-16.1)	14.6(8.7-20.5)	11.5(8.9-14.1)	9.5(7.0-11.9)	9.3(3.8-14.8)	4.4	0.001
PANSS neg	23.8(14.9-32.8)	22.3(16.1-28.5)	22.2(17.8-26.7)	15.4(12.2-18.5)	21.1(16.1-26.2)	17.1(13.5-20.7)	11.8(9.1-14.5)	11.0(4.6-16.4)	17.7	0.001
PANSS gen	36.3(23.8-48.9)	31.5(24.7-38.3)	33.7(28.4-39.1)	28.4(22.7-34.0)	29.1(21.8-36.4)	26.3(21.9-30.6)	22.8(18.6-27.0)	22.7(13.1-32.3)	19.6	0.001
PANSS total	80.5(50.6-110.4)	67.4(54.8-79.9)	75.1(62.9-87.4)	56.7(46.4-67.1)	64.8(48.4-81.2)	54.9(45.1-64.6)	44.1(36.6-51.6)	43.0(21.7-64.3)	4.4	0.001
HCR-20-H	15.0(13.5-16.5)	13.4(10.6-16.2)	14.7(11.3-18.1)	13.2(11.7-14.8)	13.6(11.8-15.5)	11.7(10.3-13.2)	11.0(9.2-12.8)	10.7(5.4-15.9)	1.8	0.099
HCR-20-C	8.3(6.4-10.3)	5.1(3.2-6.9)	6.1(4.6-7.7)	3.8(2.5-5.1)	4.8(3.2-6.4)	2.5(1.4-3.6)	1.6(0.5-2.6)	1.5(0.0-4.9)	8.9	0.001
HCR-20-R	5.7(2.7-8.6)	3.8(2.1-5.6)	3.4(2.2-4.6)	2.2(1.3-3.1)	2.7(1.4-3.9)	1.5(0.8-2.2)	1.9(1.3-2.5)	1.2(0.0-4.2)		
HCR-20-dyn	14.0(9.4-18.6)	8.9(5.5-12.4)	9.5(6.9-12.1)	6.0(3.9-8.1)	7.5(4.9-10.1)	3.9(2.3-5.6)	3.5(2.1-4.8)	2.7(0.0-9.0)	7.6	0.001
HCR-20-total	29.0(24.3-33.7)	22.3(18.6-26.0)	24.2(19.4-29.0)	19.2(16.6-21.8)	21.1(17.9-24.3)	15.7(13.7-17.7)	14.5(12.6-16.4)	13.3(1.9-24.7)	7.3	0.001
SRAMM-B	12.0(8.9-15.1)	9.0(7.2-10.8)	10.1(9.1-11.0)	10.7(9.4-12.1)	9.5(7.5-11.5)	9.5(7.6-11.4)	8.3(6.7-9.9)	9.7(4.9-14.3)	1.4	0.21
SRAMM-C	6.3(3.5-9.2)	6.2(4.3-8.1)	5.6(4.1-7.2)	4.8(3.4-6.1)	4.9(2.9-6.9)	3.6(2.8-4.4)	2.4(1.7-2.9)	2.3(0.0-6.5)	4.3	0.001
SRAMM-F	7.8(6.6-9.1)	5.3(4.2-6.4)	6.0(4.5-7.5)	6.7(5.4-7.9)	6.6(4.9-8.3)	5.1(3.9-6.2)	2.3(1.4-3.1)	2.7(0.0-6.5)	6.9	0.001
SRAMM-dyn	14.2(10.7-17.6)	11.5(9.2-13.8)	11.6(9.0-14.3)	11.5(9.4-13.7)	11.5(8.2-14.8)	8.7(7.0-10.3)	4.6(3.4-5.9)	5.0(0.0-11.7)	6.9	0.001
SRAMM-total	26.2(21.8-30.5)	20.517.9-23.1)	21.7(18.6-24.9)	22.2(19.8-24.7)	21.0(18.4-23.6)	18.1(16.2-20.1)	12.9(11.3-14.5)	14.7(4.4-24.9)	7.5	0.001
CANFOR staff rated unmet need	5.2(2.9-7.4)	3.1(1.5-4.7)	4.8(2.4-7.1)	3.1(2.1-4.1)	2.7(1.2-4.2)	1.3(0.7-1.9)	1.3(0.5-2.1)	1.0(0.0-2.3)	4.7	0.001

SABU = selective adaptive behaviour unit, intensive care; MMSU = male medium secure unit; LTLSU = longer term low secure unit; rehab. = rehabilitation pre-discharge unit. Means and 95% confidence intervals.

### Statistical methods

No data were missing. All data were entered in SPSS-18. The variable 'location at baseline' had mean = 6.47, standard deviation = 2.03, skewness = 0.008 and kurtosis = −1.041 and so could be treated as normally distributed.

Regression analysis was used to examine the antecedent covariates (predictors) of positive and of negative moves. Moves were treated as binary variables, and binary logistic regression was used with either forward or backward stepwise likelihood ratios as appropriate.

Binary logistic regression was performed first taking positive moves (compared to all others) as the dependent variable, then taking negative moves (compared to all others) as the dependent variable. Covariates were: location at baseline (a categorical variable corresponding to the 8 wards, ordered according to staff to patient ratio and place on the recovery pathway as above), DUNDRUM-1 triage security score, DUNDRUM-3 programme completion score, DUNDRUM-4 recovery score, HCR-20-H historical risk score, HCR-20-dynamic score (the sum of Clinical and Risk items), S-RAMM-B background score, S-RAMM-dynamic score (the sum of Current and Future scores), PANSS positive symptom score, PANSS negative symptom score, PANSS general symptom score, Global Assessment of Function (GAF) score and the CANFOR staff-rated unmet need score.

Predictive validity was tested using the receiver operating characteristic (ROC) area under the curve (AUC), where a significant result for the AUC is one that differs significantly from the 'random' AUC of 0.5 - where as a minimum the 95% confidence interval does not overlap 0.5.

Where analysis of variance was carried out, differences between groups can be assessed from the over-lap of confidence intervals. Univariate analysis of a general linear model was carried out for positive moves and for negative moves, co-varying for the patient's location at the beginning of the period of observation. Because the HCR-20 measure of dynamic risk is already established as a predictor of success or failure in such moves [5-8] we then co-varied for both location at baseline and the HCR-20 measure of dynamic risk (C and R items combined) to test Scott's formulation [11].

Table 1 shows that there were significant differences between units in the mean scores for the measures considered. This arises from the operational policy of placing patients according to their needs for level of therapeutic security (environmental, relational and procedural) according to assessed need [13-15]. Table 2 shows that a simple comparison of the 11 who had positive moves and 9 who had negative moves with those who had no move (n = 66) does not reveal any significant differences apart from location at baseline, with positive moves having a higher mean score for location at baseline (indicating location in more secure units) and negative moves having lower mean scores for location at baseline (indicating less secure placement at baseline). This is because those with the lowest scores (in the least secure locations) were unable to move forwards other than by discharge and conditional discharge was not legally possible during the period of this study, while those who did move forward were predominantly those moving from the more secure units such as the admission unit to the first medium secure unit or from there to the second medium secure unit. Location at baseline therefore is likely to be a significant confounder for other measures that might influence moves.

**Table 2:** Negative moves, no moves and positive moves compared (means and 95% confidence intervals)

	**Negative moves**	**No moves**	**Positive moves**	**ANOVA**	
	**N = 9**	**N = 66**	**N = 11**	**F**	**P**
Location at baseline	6.3	6.1	8.6	8.5	<0.001
	(4.8-7.8)	(5.7-6.6)	(8.1-9.2)		
DUNDRUM-1 triage security	32.4	29.6	26.6	4.1	0.02
	(29.3-35.7)	(28.5-30.8)	(24.1-29.0)		
DUNDRUM-3 programme completion	19.4	15.2	19.0	2.5	0.09
	(16.7-22.2)	(13.4-17.1)	(15.9-22.1)		
DUNDRUM-4 recovery	20.0	15.4	17.9	2.9	0.055
	(17.6-22.4)	(13.8-16.9)	(15.6-20.2)		
HCR-20-H	14.3	12.4	15.2	3.5	0.035
	(11.8-16.8)	(11.7-13.1)	(10.5-19.9)		
HCR-20-dynamic	9.7	6.3	6.9	1.9	0.148
	(6.2-13.1)	(5.1-7.5)	(3.8-10.1)		
S-RAMM-B	11.2	9.6	9.6	1.4	0.259
	(9.1-13.3)	(8.9-10.3)	(8.3-11.0)		
S-RAMM-dynamic	12.1	9.4	10.8	1.6	0.213
	(8.4-15.8)	(8.3-10.6)	(8.5-13.2)		
GAF	45.6	55.9	50.0	2.3	0.108
	(34.6-56.5)	(52.2-59.7)	(39.4-60.6)		
PANSS positive	16.7	13.6	12.6	1.02	0.364
	(11.0-22.3)	(11.9-15.3)	(8.7-16.4)		
PANSS negative	17.3	17.9	20.1	0.4	0.65
	(11.6-23.0)	(16.0-19.7)	(13.2-26.9)		
PANSS general	33.0	27.7	31.5	1.7	0.19
	(26.7-39.4)	(25.5-30.0)	(23.4-39.5)		
CANFOR staff rated unmet needs	3.9	2.7	2.6	0.9	0.40
	(1.9-5.9)	(2.0-3.3)	(1.3-4.0)		

## Results

### Participants

A total of 86 patients were assessed between February and March 2010. Six other patients were discharged during the data gathering period and did not complete the assessments and there was one further admission during the data gathering process. The six who did not complete the assessments did not differ from others in age or diagnosis. Of the 86 male patients eligible at baseline, 12 were discharged over the following 13 month period, so that the mean follow-up period was 0.98 years (SD 0.25).

### Descriptive data

The mean age was 40.6 years (SD 12.8) at baseline, mean length of stay was 7.6 years (SD 9.9). Primary diagnosis according to ICD-10 criteria [22] was schizophrenia 64 (74%), bi-polar affective disorder 9 (10%), schizoaffective disorder 7 (8%), major depressive disorder 3 (3.5%) and intellectual disability 3 (3.5%). The legal status was unfit to stand trial 8 (9%), not guilty by reason of insanity 42 (49%), prison to hospital transfer 22 (26%) and special transfer under the (civil) Mental Health Act 14 (16%).

The need for therapeutic security was assessed at baseline using the DUNDRUM-1 triage security scale [1] The mean DUNDRUM-1 score for the 86 participants was 29.5 (SD 4.8). As there are 11 items in the DUNDRUM-1 scale all scored from '0' to '4' this equates to a mean item score of 2.7, where '2' would be a score consistent with a low secure profile and '3' would be a score consistent with a medium secure profile [1,2].

### Outcome data

Table 1 shows that location at baseline accounted for significant variance in all variables except HCR-20 'H' historical scale and S-RAMM 'B' 'background' score. There were 11 positive moves and 9 negative moves with no individual having both a positive and a negative move (*X*2 = 1.5, df = 1, p = 0.225). Table 2 shows that when negative moves, no moves and positive moves were compared, there were few significant differences overall, with only the location at baseline, DUNDRUM-1 triage security measure and HCR-20-H historical measure reaching significance.

### Binary logistic regression

Because location at baseline is likely to be confounding the differences due to risk and need for therapeutic security, binary logistic regression was performed using forwards selection and the likelihood ratio.

A positive move was the dependent variable (compared to all others) and covariates were location at baseline, DUNDRUM-1, DUNDRUM-3, DUNDRUM-4, HCR-20-H, HCR-20-dynamic, S-RAMM-B, S-RAMM-dynamic, GAF, PANSS positive, PANSS negative, PANSS general, CANFOR-staff-rated unmet need. By step 4, the iterative process had concluded with location at baseline (odds ratio 16.3, 95% confidence interval 2.6 - 102.7, p = 0.003 indicating that more secure places were more likely to have a positive move), DUNDRUM-1 triage security (odds ratio 0.595, 95% CI 0.394-0.900, p = 0.014 indicating that those with higher need for therapeutic security were less likely to have a positive move), HCR-20-dynamic (odds ratio 0.410, 95% CI 0.204-0.821, p = 0.012 indicating that those with higher dynamic risk scores were less likely to have a positive move) and PANSS general symptom score (odds ratio 1.257, 95% CI 1.015-1.556, p = 0.036 indicating that those with higher general symptoms were more likely to have a positive move) emerging as significant. The same result was obtained using backwards stepwise selection (likelihood ratio).

When a negative move was taken as the dependent variable (compared to all others) using backward selection (likelihood ratio) starting with the same set of variables the iteration concluded at step 10 and the remaining covariates were location at baseline (odds ratio 0.441 95% confidence interval 0.218-0.892, p = 0.023 indicating that those in more secure units were less likely to have a negative move), DUNDRUM-3 programme completion (odds ratio 1.268, 95% CI 1.034-1.556, p = 0.023 indicating that those who had made less progress in treatment were more likely to have a negative move), HCR-20-dynamic (odds ratio 1.479, 95% CI 1.100-1.989, p = 0.01 indicating that higher dynamic risk scores were more likely to have negative moves) and PANSS negative score (odds ratio 0.800, 95% CI 0.667-0.959, p = 0.016 showing a small protective effect for negative symptoms). Comparing this with the model obtained for positive moves, a model for negative moves could be constructed using binary regression backward selection (likelihood ratio) in which only the three expected terms were significant - location at baseline (odds ratio 0.567, 95% CI 0.327-0.984, p = 0.044 with more secure places being less likely to lead to negative moves as before), DUNDRUM-1 triage security (odds ratio 1.224, 95% CI 1.012-1.482, p = 0.038 indicating that those with higher need for therapeutic security were more likely to have a negative move) and HCR-20 dynamic score (odds ratio 1.270, 95% CI 1.049-1.538, p = 0.014 indicating that higher dynamic risk scores led to negative moves) and no other variable, if added was retained in the equation.

### Receiver operating characteristic

Because of the likely confounding effect of location at baseline, and because it was inherently not possible for those in the pre-discharge wards to have positive moves or those in the most secure wards to have negative moves, the receiver operating characteristic was unlikely to be capable of detecting the predictive effect of most measures.

In spite of this, for positive moves both location at baseline (area under the curve AUC = 0.861, (95% confidence interval (CI) 0.779-0.943, p < 0.001) and DUNDRUM-1 triage security score (AUC = 0.281 (95% CI 0.148-0.413 p = 0.019) were significantly better than random prediction indicating that those in more secure units were more likely to have positive moves and those with higher (worse) scores on the DUNDRUM-1 were less likely to have positive moves but no other measure differed significantly from the random area under the curve.

For negative moves, larger (more problematic) scores tended to predict negative moves though location at baseline was not significant (AUC = 0.486, 95% CI 0.297-0.674, p = 0.888) while the DUNDRUM-1 triage security score (AUC = 0.696, 95% CI 0.536-0.855, p = 0.056), DUNDRUM-3 programme completion (AUC = 0.643, 95% CI 0.509-0.870, p = 0.163), DUNDRUM-4 recovery (AUC = 0.719, 95% CI 0.569-0.870, p = 0.032), PANSS general symptoms (AUC = 0.686, 95% CI 0.521-0.852, p = 0.069), HCR-20-dynamic score (AUC = 0.702, 95% CI 0.535-0.869, p = 0.048) and GAF (AUC = 0.317, 95% CI 0.141-0.494, p = 0.074) were all marginally significant (95% confidence intervals did not overlap the random value for the area under the curve of 0.5). The DUNDRUM-4 recovery score and HCR-20 dynamic scores were the best of these. No other measure differed significantly from the random area under the curve.

### Secondary analysis: analysis of variance for positive moves

Eleven patients had positive moves. Table 3 shows that the 'crude' mean DUNDRUM-1 triage security score was significantly lower for those who had subsequent positive moves, but all other measures were not significantly different except for the HCR-20 'H' score which was higher for those who had moves than for those who did not. The tendency for most variables to have higher 'crude' means for those who had positive moves is explained by the effect of location at baseline, since adjusting for this reveals marginal means that were significantly lower (better) for those who had subsequent positive moves for the DUNDRUM-1 (F = 14.2,df = 1, p < 0.001), DUNDRUM-3 (F = 5.4, p = 0.022), DUNDRUM-4 (F = 5.7, p = 0.02), GAF (F = 4.6, p = 0.034), PANSS positive score (F = 8.4, p = 0.005), HCR-20 'C' (F = 10.8 p = 0.002) 'R' (F = 4.1 p = 0.047) and dynamic (sum of 'C' and 'R' scores F = 8.6, p = 0.004), S-RAMM total (F = 4.3, p = 0.042) score and the CANFOR staff-rated unmet needs (F = 6.7, p = 0.012). Because the HCR-20 dynamic score is known to be an influence on such moves and shows as a powerful influence in this analysis, UniAnova with positive moves as fixed factor was repeated co-varying for location at baseline and HCR-20 dynamic ('C' + 'R') score. In this analysis all the variables which were significantly associated with subsequent positive moves when correcting for location at baseline were no longer significant, except for the DUNDRUM-1 triage security scale (corrected model F = 7.3 df = 3 p < 0.001, location at baseline F = 13.8 df = 1 p < 0.001, HCR-20 dynamic score F = 1.6 df = 1 NS, DUNDRUM-1/positive moves F = 15.9 df = 1 p < 0.001).

**Table 3:** Positive moves controlling for location and for HCR-dynamic risk scores

	**'crude' means (SD)**			**Marginal means (SE) adjusted for location at baseline**			**Marginal means (SE) adjusted for location at baseline and HCR-20 dynamic (C + R) scores**		
	**No Move**	**Positive move**	**ANOVA F/p**	**No Move**	**Positive move**	**ANOVA F/p**	**No move**	**Positive move**	**ANOVA F/p**
**n**	**75**	**11**	**Df = 1**	**75**	**11**	**Df = 1**	**75**	**11**	**Df = 1**
DUNDRUM-1 triage security	29.9(4.8)	26.6(3.7)	5.2/0.026	30.3(0.51)	24.5(1.42)	14.2/0.001	30.4(0.5)	23.9(1.5)	15.9/0.001
DUNDRUM-3 programme completion	15.8(7.3)	19.0(4.7)	2.0/0.158	16.7(0.55)	12.8(1.53)	5.4/0.022	16.5(0.5)	14.1(1.5)	2.0/0.160
DUNDRUM-4 Recovery	15.9(7.3)	17.9(3.4)	1.0/0.312	16.7(0.51)	13.0(1.42)	5.7/0.02	16.5(0.5)	14.3(1.4)	2.0/0.157
GAF	54.7(15.4)	50.0(15.8)	0.9/0.352	52.9(1.37)	61.8(3.8)	4.6/0.034	54.1(0.9)	54.2(2.8)	0.01/0.9
PANSS pos	14.0(6.9)	12.6(5.8)	0.4/0.51	14.6(0.7)	8.6(1.9)	8.4/0.005	14.1(0.5)	12.0(1.5)	1.5/0.222
PANSS neg	17.8(7.4)	20.1(10.2)	0.8/0.36	18.4(0.8)	15.9(2.3)	1.1/0.304	17.9(0.7)	19.4(1.9)	0.5/0.498
PANSS gen	28.4(9.3)	31.5(12.0)	0.9/0.3	29.0(1.0)	26.9(2.9)	0.4/0.520	28.3(0.8)	31.9(2.4)	1.9/0.171
PANSS total	60.2(21.4)	64.1(25.8)	0.3/0.6	62.0(3.2)	51.4(6.3)	2.4/0.123	60.3(1.7)	63.3(4.8)	0.3/0.563
HCR-20-H	12.6(3.0)	15.2(6.9)	4.7/0.034	12.7(0.4)	14.1(1.2)	0.95/0.331	12.6(0.4)	14.8(1.2)	2.6/0.138
HCR-20-C	4.1(3.0)	4.1(2.9)	0.0/0.9	4.4(0.3)	1.8(0.7)	11.2/0.001	n/a	n/a	n/a
HCR-20-R	2.6(2.2)	2.8(2.1)	0.1/0.8	2.8(0.2)	1.5(0.6)	3.8/0.054	n/a	n/a	n/a
HCR-20-dyn	6.7(4.9)	6.9(4.7)	0.0/0.9	7.2(0.4)	3.3(1.2)	8.6/0.004	n/a	n/a	n/a
HCR-20-total	19.3(6.8)	22.1(8.7)	1.5/0.2	19.9(0.7)	17.3(1.9)	1.8/0.184	19.3(0.4)	21.5(1.2)	2.3/0.135
SRAMM-B	9.8(2.9)	9.6(2.1)	0.0/0.9	9.9(0.3)	8.9(0.9)	0.9/0.339	9.9(0.3)	8.9(0.9)	0.7/0.405
SRAMM-C	4.4(2.5)	5.1(3.1)	0.6/0.4	4.7(0.3)	3.5(0.7)	2.2/0.142	4.5(0.2)	5.0(0.5)	0.9/0.321
SRAMM-F	5.3(2.8)	5.7(1.6)	0.2/0.6	5.5(0.3)	4.2(0.8)	2.5/0.115	5.4(0.3)	4.9(0.8)	0.4/0.540
SRAMM-dyn	9.8(4.8)	10.8(3.5)	0.5/0.5	10.2(0.5)	7.7(1.3)	3.3/0.072	9.8(0.4)	9.9(1.0)	0.0/0.9
SRAMM total	19.5(5.9)	20.5(4.1)	0.3/0.6	20.1(0.6)	16.6(1.5)	4.3/0.042	19.7(0.5)	18.9(1.4)	0.3/0.582
CANFOR staff-rated unmet need	2.8(2.7)	2.6(2.1)	0.0/0.8	3.0(0.3)	1.0(0.7)	6.7/0.012	2.9(0.2)	1.6(0.7)	2.8/0.1

'Crude' data - means and standard deviations. Marginal means (and standard errors) from univariate analysis of variance co-varying for the key confounders - first location at baseline, then both location and HCR-20 dynamic score.

### Secondary analysis: analysis of variance for negative moves

Table 4 shows the same tests for the associations of subsequent negative moves. Nine patients had negative moves. Crude data indicate higher scores for almost all measures with this reaching statistical significance for DUNDRUM-1, DUNDRUM-4, HCR-20-R, HCR-20 total score and S-RAMM total score. Co-varying for location at baseline, the marginal means were significantly higher (worse) for those having subsequent negative moves for DUNDRUM-1 (F = 4.3 p = 0.042), DUNDRUM-3 (F = 6.2 p = 0.015), DUNDRUM-4 (F = 9.1 p = 0.003), GAF (significantly lower, better scores for negative moves F = 6.4 p = 0.013), and significantly higher, worse scores for HCR-20 'C' (F = 4.8 p = 0.03), HCR-20 'R' (F = 6.9 p = 0.01), HCR-dynamic ('C' + 'R' F = 6.8 p = 0.011), HCR-20 total score (F = 7.1 p = 0.009), S-RAMM-'C' score (F = 5.9 p = 0.017) and S-RAMM total score (F = 7.2 p = 0.009).

**Table 4:** Negative moves controlling for location and for HCR-dynamic risk scores

	**'crude' means (SD)**			**Marginal means (SE) adjusted for location at baseline**			**Marginal means (SE) adjusted for location at baseline and HCR-20 dynamic (C + R) scores**		
	**No Move**	**Negative move**	**ANOVA**	**No Move**	**Negative move**	**ANOVA**	**No move**	**Negative move**	**ANOVA**
**N**	**77**	**9**	**Df = 1**	**77**	**9**	**Df = 1**	**77**	**9**	**Df = 1**
DUNDRUM-1 triage security	29.2(4.8)	32.4(4.2)	3.8/0.053	29.2(0.5)	32.5(1.5)	4.3/0.042	29.2(0.5)	32.8(1.6)	4.7/0.034
DUNDRUM-3 programme completion	15.8(7.3)	19.4(3.6)	2.2/0.144	15.8(0.5)	19.8(1.5)	6.2/0.015	15.9(0.5)	18.6(1.5)	2.9/0.09
DUNDRUM-4 Recovery	15.8(6.1)	20.0(3.1)	4.2/0.043	15.8(0.5)	20.3(1.4)	9.1/0.003	15.9(0.5)	19.2(1.4)	5.2/0.025
GAF	55.1(15.4)	45.6(14.2)	3.1/0.08	55.1(1.3)	44.9(3.9)	6.4/0.013	54.3(0.9)	51.7(2.8)	0.8/0.375
PANSS pos	13.5(6.6)	16.7(7.3)	1.8/0.181	13.5(0.7)	16.9(2.0)	2.6/0.114	13.8(0.5)	13.5(1.6)	0.0/0.86
PANSS neg	18.2(7.9)	17.3(7.4)	0.1/0.76	18.1(0.8)	17.6(0.3)	0.1/0.823	18.5(0.6)	14.2(1.9)	4.3/0.041
PANSS gen	28.3(9.7)	33.0(8.3)	1.9/0.167	28.2(1.0)	33.3(2.9)	2.6/0.109	28.7(0.8)	29.0(2.5)	0.0/0.9
PANSS total	59.9(21.9)	67.0(21.7)	0.8/0.362	59.8(2.2)	67.7(6.5)	1.3/0.249	61.1(1.6)	56.8(4.9)	0.6/0.4
HCR-20-H	12.7(3.8)	14.3(3.2)	1.5/0.242	12.7(0.4)	14.4(1.2)	1.8/0.188	12.8(0.4)	14.0(1.2)	0.9/0.3
HCR-20-C	3.9(2.9)	5.6(3.0)	2.6/0.116	3.9(0.3)	5.7(0.8)	4.8/0.031	n/a	n/a	n/a
HCR-20-R	2.5(2.2)	4.1(1.6)	4.7/0.03	2.5(0.2)	4.2(0.6)	7.0/0.010	n/a	n/a	n/a
HCR-20-dyn	6.4(4.8)	9.7(4.5)	3.8/0.055	6.4(0.4)	9.9(1.3)	6.8/0.011	n/a	n/a	n/a
HCR-20-total	19.1(6.9)	24.0(7.1)	4.0/0.048	19.1(0.6)	24.3(1.9)	7.1/0.009	19.5(0.4)	20.8(1.2)	n/a
SRAMM-B	9.6(2.8)	11.2(2.7)	2.8/0.099	9.6(0.3)	11.3(0.9)	3.0/0.087	9.6(0.3)	11.2(0.9)	2.7/0.108
SRAMM-C	4.3(2.5)	6.1(2.8)	3.9/0.053	4.3(0.3)	6.2(0.7)	5.9/0.017	4.8(0.2)	4.9(0.5)	0.6/0.455
SRAMM-F	5.3(2.7)	6.0(2.7)	0.6/0.451	5.3(0.3)	6.1(0.8)	0.9/0.334	5.4(0.3)	5.4(0.8)	0.0/0.9
SRAMM-dyn	9.6(4.6)	12.1(4.8)	2.3/0.132	9.6(0.4)	12.3(1.3)	3.9/0.051	9.8(0.3)	10.3(1.0)	0.2/0.7
SRAMM total	19.2(5.5)	23.3(6.4)	4.4/0.039	19.2(0.5)	23.6(1.5)	7.2/0.009	19.4(0.5)	21.5(1.4)	2.1/0.15
CANFOR staff-rated unmet need	2.7(2.6)	3.9(2.6)	1.9/0.175	2.6(0.3)	3.9(0.8)	2.8/0.098	2.7(0.3)	3.4(0.7)	0.6/0.4

'Crude' data - means and standard deviations. Marginal means (and standard errors) from univariate analysis of variance co-varying for the key confounders - first location at baseline and then both location and HCR-20 dynamic score.

Finally, correcting for both location at baseline and HCR-20 dynamic score eliminated all but the DUNDRUM-1 (corrected model F = 3.3 df = 3 p = 0.024, location at baseline F = 5.1 df = 1 p = 0.027, HCR-20 dynamic score F = 0.4 df = 1 NS, DUNDRUM-1/negative moves F = 4.7 df = 1 p = 0.034). The only other association with subsequent negative moves when co-varying for location at baseline and HCR-20 dynamic score was the PANSS negative symptom scale, with a significantly lower marginal mean (corrected model F = 26.1 df = 3 p < 0.001, location at baseline F = 0.4 df = 1 NS, HCR-20 dynamic score F = 43.6 df = 1 p < 0.001, PANSS negative/negative moves F = 4.3 df = 1 p = 0.041).

Adding the DUNDRUM-1 to the list of co-variants eliminated PANSS negative symptoms while leaving location at baseline as significant.

## Discussion

### Key results

Binary logistic regression indicated that for positive moves the location at baseline, DUNDRUM-1 triage security along with the HCR-20-dynamic risk score and PANSS general symptoms scores were associated with subsequent positive moves. The receiver operating characteristic could not be corrected for location at baseline, but the DUNDRUM-1 triage security score at baseline was still associated with subsequent positive moves. When ANOVA was used to adjust for location, the DUNDRUM-1 triage security scale, the DUNDRUM-3 programme completion scale and the DUNDRUM-4 recovery scale were significantly lower (nearer to recovery) for those who subsequently had positive moves i.e. from higher to lower secure wards. The same was true for the GAF, PANSS positive score, HCR-20 dynamic score, S-RAMM total and CANFOR staff-rated unmet need. When further adjusted for both location at baseline and the HCR-20 dynamic score however, only the DUNDRUM-1 triage security score remained significantly associated with subsequent positive moves indicating that the DUNDRUM-1 and HCR-20 dynamic scores between them accounted for most of the variance. The DUNDRUM-1 triage security score was significantly associated with subsequent positive moves using all three analyses while the HCR-20 dynamic score was significantly associated with subsequent positive moves in two of three analyses.

Binary logistic regression indicated that the significant associations with subsequent negative moves were location at baseline, DUNDRUM-3 programme completion score, HCR-20 dynamic score and PANSS negative symptom scores. However a model could also be constructed in which location at baseline, DUNDRUM-1 and HCR-20 dynamic scores together were the only significant associations with subsequent negative moves. The receiver operating characteristic for negative moves was significantly better than random for DUNDRUM-1, DUNDRUM-3, DUNDRUM-4, HCR-20 dynamic score, PANSS general score and GAF score, though some were marginal. Analysis of variance correcting for location at baseline showed the DUNDRUM-1 triage security scale, DUNDRUM-3 programme completion and DUNDRUM-4 recovery scales were significantly higher (further from recovery) for those who went on to have negative moves (i.e. from less secure to more secure wards) while the GAF was lower (further from recovery), HCR-20 dynamic and total scores, S-RAMM 'C' and S-RAMM total scores were higher (worse). When further adjusted for both location at baseline and the HCR-20 dynamic measure, the DUNDRUM-1 triage security score remained significantly higher (worse) in those who had negative moves along with a significantly lower (better) PANSS negative symptom score. Like the determinants of positive moves, the DUNDRUM-1 and HCR-20 dynamic scores were consistent predictors, while the DUNDRUM-3 and DUNDRUM-4 appeared to be consistently relevant also. We interpret this as indicating that the DUNDRUM-1 triage security instrument measures that 'seriousness' or 'gravity' of risk referred to by Scott [11] as a complimentary factor to risk and which is largely independent of risk. The DUNDRUM-3 programme completion and DUNDRUM-4 recovery measures are not completely independent of risk but do assess what clinicians appear to believe to be progress in the amelioration of both risk and the seriousness of risk.

### Limitations

Although this is a true prospective cohort study, it is a naturalistic observational study. Positive and negative moves, the outcome measures, were not decided on as a result of any protocol and so may have been decided inconsistently. Such moves are however factual and are real outcome measures. Positive moves were decided on by clinicians on the basis of individual clinical assessments in response to the availability of places in less secure units. Negative moves were decided in response to individual crises and cumulative assessments. Since the object of this study was to examine these clinical decision making processes as in a previous study [4], this is both a weakness and the object of the study.

### Interpretation

These data add further weight to the validity of the DUNDRUM-1 as a measure of perceived need for therapeutic security in practice [2,3]. The complimentary relationship of the DUNDRUM-1 with the HCR-20 dynamic measures of risk of violence is interesting. The HCR-20 'H' historical sub-scale was a notably poor predictor of these outcomes. Clinicians make decisions based on a combination of current clinical risk and future risk rather than static historical predictors of risk, but retain a significant degree of caution based on the seriousness of the risk. this resembles Scott's 1977 formula for perceived 'dangerousness' as the balancing of risk and seriousness or 'gravity' of the risk [11]. These two factors appear to outweigh other measures. However the DUNDRUM-3 programme completion and DUNDRUM-4 recovery scales are significantly related to positive and negative moves when dynamic risk is not taken into account. This reflects their strong correlation with the HCR-20 and it's component sub-scales [4]. The DUNDRUM-3 and DUNDRUM-4 are substantially different from the HCR-20 items in their content and therefore continue to fulfill an essential function in measuring progress in treatments relevant to risk and the seriousness of the risk, and measures of recovery in a forensic context that are relevant to the clinician's willingness to take therapeutic risks such as allowing accompanied and unaccompanied leave outside the secure hospital [4].

The role of suicide risk (measured by the S-RAMM) was surprisingly modest but this may reflect the strong correlation between the S-RAMM and the HCR-20 [13,15]. Better GAF scores were significantly associated with subsequent progress as were lower positive symptom scores, while worse GAF scores and fewer negative symptoms were associated with subsequent negative moves.

### Generalisability

This study shows that the DUNDRUM-1 triage security score and the HCR-20 dynamic risk measures were associated with subsequent positive and negative moves. The DUNDRUM-3 programme completion and DUNDRUM-4 recovery instruments were also relevant. As structured professional judgement instruments, it is the item content of these instruments that is of greatest potential practical value to clinicians. This will form the basis of future studies. It remains to be seen if the DUNDRUM Toolkit instruments predict moves to the community - this is currently the subject of another prospective study. Many existing studies have shown that the HCR-20 predicts violence and recidivism, and the more clinically useful outcome measure of a negative move from the community back to a secure hospital [5]. The interaction between 'pure' risk as measured by the HCR-20 and seriousness of the risk, as reflected by need for therapeutic security (DUNDRUM-1) is in keeping with one of the oldest theoretical formulations in forensic mental health [11] and might be expected to generalise to such decisions but requires a prospective study to test this.

## Conclusions

The DUNDRUM-1 triage security scale and HCR-20 dynamic risk score ('C' and 'R' items) predicted positive and negative moves between levels of therapeutic security. It appears that clinicians base their decisions on both the current indicators of risk of violence and the need for therapeutic security based on the seriousness of past behaviours. It would appear that in practice clinicians are applying the same formula described by Scott [11] for perceived 'dangerousness', balancing risk and gravity. The DUNDRUM-3 programme completion scale, a measure of treatment engagement and success, and the DUNDRUM-4 recovery scale were predictors of positive and negative moves, though unlike the DUNDRUM-1 they did not add any statistical power to the HCR-20 dynamic score. However in the same way that the content of the HCR-20 is now regarded as more important than the overall score in guiding risk management and treatment, the content of the DUNDRUM-3 and DUNDRUM-4 is so different from the HCR-20 as to make their use valuable when making structured professional judgements, when communicating and explaining decisions and when planning further treatment. The DUNDRUM-3 and DUNDRUM-4 also offer a means of changing practice and looking objectively at patients' current progress in a way that may offset the 'halo' effect of the historical 'seriousness' or gravity of past behaviour.

## Competing interests

The authors declare that they have no competing interests. This study received no external or special funding as it was carried out as part of an audit of service effectiveness.

## Authors’ contributions

MD rated patients using the DUNDRUM-1, DUNDRUM-3 and DUNDRUM-4, revised drafts of the article and collated the database with ZA; LN and OG rated patients using the PANSS and GAF. ED and KMcD coordinated and collated ratings of HCR-20 and S-RAMM. KMcD collated the CANFOR ratings and contributed to the DUNDRUM-4. ED helped coordinate the rating of the DUNDRUM-3. SM advised on aspects of the data analysis. HGK wrote the first draft of the handbook, designed the study and carried out the data analysis with MD. All contributed to the authorship of the paper. All authors read and approved the final manuscript.

## Pre-publication history

The pre-publication history for this paper can be accessed here:

http://www.biomedcentral.com/1471-244X/12/80/prepub
